# Hematological Correlates of Hemoglobinopathies in Women of Reproductive Age With Anemia: A High-Performance Liquid Chromatography (HPLC)-Based Cross-Sectional Study

**DOI:** 10.7759/cureus.111733

**Published:** 2026-06-29

**Authors:** Shanti Marandi, Manoj K Paswan, Anshu Jamaiyar, Deepali Tirkey, Sunil Kumar Mahto

**Affiliations:** 1 Pathology, Rajendra Institute of Medical Sciences, Ranchi, IND

**Keywords:** anemia, hemoglobinopathies, hplc, red cell indices, sickle cell disease, β-thalassemia

## Abstract

Background: Anemia continues to pose a significant public health challenge, particularly among women of reproductive age. Despite ongoing nutritional intervention programs and iron supplementation strategies, the burden of anemia remains persistently high. In addition to iron deficiency anemia (IDA), inherited hemoglobin disorders such as β-thalassemia and sickle cell disease are important contributors to anemia. High-performance liquid chromatography (HPLC) has emerged as a reliable and widely used method for the identification of hemoglobin variants and the quantification of hemoglobin A₂ (HbA₂) and hemoglobin F (HbF). The objective of our study was to investigate the range of hemoglobinopathies identified by HPLC and to compare them with routine hematological parameters.

Methods: Our study was conducted in the Department of Pathology, Rajendra Institute of Medical Sciences, Ranchi, over 18 months from August 2024 to January 2026. A total of 277 anemic females aged 19-49 years with hemoglobin levels below 11 g/dL were enrolled. Hematological parameters were analyzed using an automated hematology analyzer, while hemoglobin fraction analysis was performed using cation-exchange HPLC. Statistical analysis was performed using IBM SPSS Statistics for Windows, Version 27 (Released 2019; IBM Corp., Armonk, New York, United States).

Results: The mean age of participants was 33.2 ± 9.07 years, and the mean hemoglobin level was 9.7 ± 0.95 g/dL. Microcytic hypochromic anemia was the predominant pattern with elevated red cell distribution width (RDW) values indicating anisocytosis. HPLC analysis revealed abnormal hemoglobin patterns in 44% of participants. Sickle cell trait was the most common abnormality (26%), followed by sickle cell disease (11%) and β-thalassemia trait (7%). RDW showed significantly superior discriminative ability for predicting hemoglobinopathies compared to mean corpuscular volume (MCV) and mean corpuscular hemoglobin (MCH).

Conclusion: Hemoglobinopathies were common among anemic women of reproductive age, with sickle cell trait being the predominant abnormality detected on HPLC analysis. Routine hematological parameters showed considerable overlap, whereas RDW demonstrated better discriminatory ability but was insufficient alone. HPLC improved the detection and characterization of hemoglobin variants and may be useful in the targeted evaluation of high-risk populations.

## Introduction

Anemia is still a major public health problem in India, especially among women of reproductive age, where it affects adverse pregnancy outcomes, increased maternal morbidity, reduced physical performance, and work productivity [1-3]. Ongoing community-based interventions for the prevention of anemia through nutritional intervention programs and iron supplementation strategies have so far not been effective in controlling the burden of anemia, indicating that non-iron-deficiency factors are also potentially significant contributory factors. The reproductive-age women are particularly susceptible due to menstrual blood loss, pregnancy, lactation, nutritional deficiencies, and socio-economic determinants [4]. In addition to iron deficiency, deficiencies of vitamin B12 and folate are also recognized contributors to anemia among women of reproductive age and may influence hematological parameters. Iron deficiency has been analyzed in more detail as the predominant causative factor; nevertheless, a large number of patients present with recurrent anemia for no obvious reason and may sustain underlying genetic disorders [5]. Apart from iron deficiency anemia (IDA), hereditary hemoglobin disorders, such as β-thalassemia and sickle cell disease, also contribute significantly to anemia in India, especially in eastern and tribal populations, where recessive hemoglobinopathies are frequently identified [6]. In reality, these disorders can often have microcytic hypochromic blood pictures that overlap with IDA, making it difficult to distinguish between these conditions based on routine hematological parameters alone [7,8].

Routine blood indices such as mean corpuscular volume (MCV), mean corpuscular hemoglobin (MCH), and red cell distribution width (RDW) provide useful preliminary diagnostic clues in the evaluation of anemia. However, considerable overlap exists between IDA and hemoglobinopathies, especially β-thalassemia trait, thereby limiting the specificity of these parameters when used independently [8].

High-performance liquid chromatography (HPLC) has emerged as a reliable and widely used method for the identification of hemoglobin variants and the quantification of hemoglobin A₂ (HbA₂) and hemoglobin F (HbF), thereby improving diagnostic accuracy for hemoglobinopathies [9]. Several studies have demonstrated the utility of HPLC in detecting clinically significant hemoglobin variants among anemic individuals [10]. Limited data exist regarding the frequency and hematological correlation of hemoglobinopathies among anemic reproductive-age women in eastern India. The objective of our study was to investigate the range of hemoglobinopathies identified by HPLC and to compare them with routine hematological parameters.

## Materials and methods

Our study was conducted as a cross-sectional observational study in the Department of Pathology, Rajendra Institute of Medical Sciences, Ranchi. Our study had a duration of 18 months from August 2024 to January 2026. Ethical clearance was obtained from the Institutional Ethics Committee (Letter No. 133 dated 08.02.2025), and written informed consent was obtained from all participants before enrollment.

Anemia severity was classified according to WHO criteria as mild (hemoglobin 10.0-10.9 g/dL), moderate (7.0-9.9 g/dL), and severe (<7.0 g/dL) [11]. We included anemic females aged 19-49 years with hemoglobin levels below 11 g/dL who were referred for hemoglobin analysis. Patients with a history of recent blood transfusion, those unwilling to participate, individuals outside the target age group, and patients with known comorbid conditions were excluded to minimize confounding factors. Based on a previously reported frequency of abnormal hemoglobin patterns of approximately 20% among anemic patients, with 5% precision, a 95% confidence interval, and a 10% non-response rate, using the formula $n = Z&sup2;pq/d&sup2;$, a minimum sample size of 271 was estimated [12]. A total of 277 participants fulfilling the inclusion criteria were eventually enrolled using a convenience sampling method. Venous blood samples were collected under aseptic precautions in ethylenediaminetetraacetic acid (EDTA) vials for complete blood count and HPLC analysis. An abnormal hemoglobin pattern was defined as any chromatographic profile showing abnormal hemoglobin fractions or values outside the reference ranges. HbA₂ values >3.5% were considered suggestive of the β-thalassemia trait. Sickle cell trait was diagnosed by the presence of HbS with predominant HbA, whereas sickle cell disease was diagnosed by predominant HbS with absent or markedly reduced HbA. β-thalassemia major was diagnosed based on markedly elevated HbF with absent or severely reduced HbA. Hematological parameters, including hemoglobin concentration, red blood cell count, hematocrit, MCV, MCH, MCH concentration (MCHC), and RDW, were analyzed using an automated hematology analyzer. Hemoglobin fraction analysis was performed using cation-exchange HPLC (Bio-Rad Variant II, Bio-Rad Laboratories, USA) according to manufacturer protocols. Serum ferritin, serum iron, total iron binding capacity, vitamin B12, and folate levels were not evaluated as part of the study protocol.

We performed statistical analysis using IBM SPSS Statistics for Windows, Version 27 (Released 2019; IBM Corp., Armonk, New York, United States). Continuous variables were expressed as mean ± standard deviation, while categorical variables were presented as frequencies and percentages. Group comparisons were performed using the chi-square test or Fisher’s exact test, wherever appropriate. Pearson correlation analysis was used to evaluate associations between hematological indices and hemoglobin fractions. Receiver operating characteristic (ROC) curve analysis was performed to assess the diagnostic performance of red cell parameters. A p-value of <0.05 was considered statistically significant.

## Results

Baseline characteristics of the study population

We included 277 anemic females aged 19-49 years, with a mean age of 33.2 ± 9.07 years. The mean hemoglobin level was 9.7 ± 0.95 g/dL. Hematological evaluation predominantly showed a microcytic hypochromic pattern, with a mean MCV of 77.8 ± 9.55 fL and a mean MCH of 25.3 ± 4.07 pg. Elevated RDW values (16.8 ± 3.58%) indicated significant anisocytosis. Baseline hematological and HPLC parameters are summarized in Table 1.

**Table 1:** Baseline characteristics of study population RBC: red blood cell; hematocrit: packed cell volume; MCV: mean corpuscular volume; MCH: mean corpuscular hemoglobin; MCHC: mean corpuscular hemoglobin concentration; RDW: red cell distribution width; HbA: hemoglobin A; HbA₂: hemoglobin A₂; HbF: fetal hemoglobin; HbS: hemoglobin S

Parameter	Mean ± SD
Age (years)	33.2 ± 9.07
Hemoglobin (g/dL)	9.7 ± 0.95
RBC (million/µL)	3.8 ± 0.55
Hematocrit (%)	29.9 ± 3.35
MCV (fL)	77.8 ± 9.55
MCH (pg)	25.3 ± 4.07
MCHC (g/dL)	32.9 ± 2.03
RDW (%)	16.8 ± 3.58
HbA (%)	75.3 ± 28.69
HbA₂ (%)	2.8 ± 0.73
HbF (%)	4.2 ± 7.07
HbS (%)	16.6 ± 23.31

Age distribution and severity of anemia

Most participants belonged to the 19-30 years age group, accounting for 111 (40%), followed by 95 (34%) participants in the 31-40 years group and 71 (26%) participants in the 41-49 years group. Mild anemia was the predominant presentation in 226 (82%) participants, whereas moderate and severe anemia were observed in 45 (16%) and six (2%) participants, respectively (Table 2).

**Table 2:** Age distribution and severity of anemia

Variable	Category	N (%)
Age Group	19–30 years	111 (40%)
	31–40 years	95 (34%)
	41–49 years	71 (26%)
Severity of Anemia	Mild	226 (82%)
	Moderate	45 (16%)
	Severe	6 (2%)

Distribution of hemoglobinopathies

**Figure 1:**
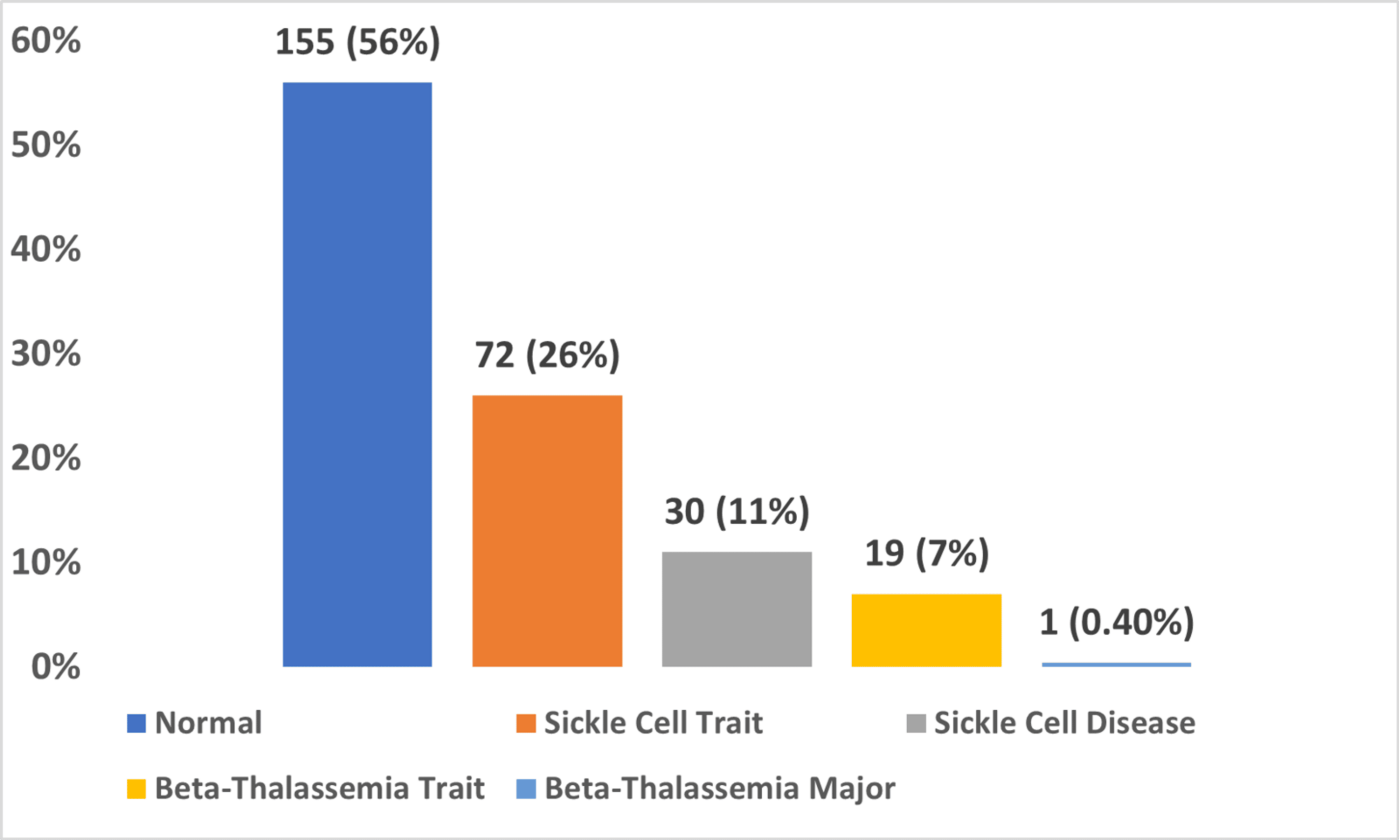
Distribution of hemoglobinopathies based on high-performance liquid chromatography (HPLC)

HPLC analysis revealed abnormal hemoglobin patterns in 122 (44%) participants, whereas normal HPLC profiles were observed in 155 (56%). Sickle cell trait was the most common abnormality, identified in 72 (26%) participants, followed by sickle cell disease in 30 (11%) and β-thalassemia trait in 19 (7%) participants. Only one case of β-thalassemia major was identified (Figure 1).

Comparison of hematological and HPLC parameters across hemoglobinopathy subgroups

Subgroup analysis demonstrated significant differences in hematological and chromatographic parameters across individual hemoglobinopathy categories (sickle cell trait, sickle cell disease, and beta thalassemia trait). RDW, HbA₂, HbF, and HbS levels differed significantly across most subgroups, whereas conventional red cell indices showed variable discriminatory ability (Table 3).

**Table 3:** Comparison of hematological and HPLC parameters across hemoglobinopathy subgroups SCT: sickle cell trait; SCD: sickle cell disease; BTT: β-thalassemia trait; HPLC: high-performance liquid chromatography; NS: not significant; RBC: red blood cell; hematocrit: packed cell volume; MCV: mean corpuscular volume; MCH: mean corpuscular hemoglobin; MCHC: mean corpuscular hemoglobin concentration; RDW: red cell distribution width; HbA: hemoglobin A; HbA₂: hemoglobin A₂; HbF: fetal hemoglobin; HbS: hemoglobin S

Parameter	SCT vs Non-SCT (p-value)	SCD vs Non-SCD (p-value)	BTT vs Non-BTT (p-value)
Hemoglobin	0.214 (NS)	<0.001	0.039
RBC	0.487 (NS)	0.318 (NS)	<0.001
Hematocrit	0.156 (NS)	<0.001	<0.001
MCV	0.624 (NS)	0.281 (NS)	<0.001
MCH	0.392 (NS)	0.447 (NS)	0.015
MCHC	0.518 (NS)	0.073 (NS)	<0.001
RDW	0.001	<0.001	0.026
HbA	<0.001	<0.001	0.439 (NS)
HbA₂	<0.001	<0.001	<0.001
HbF	0.571 (NS)	<0.001	0.663 (NS)
HbS	<0.001	<0.001	—

Comparison between the normal and hemoglobinopathy groups

Participants with hemoglobinopathies had significantly lower hemoglobin and hematocrit levels and significantly higher RDW values compared to those with normal HPLC patterns. Significant differences were also observed in HbA, HbA₂, HbF, and HbS fractions (Table 4).

**Table 4:** Comparison between normal and hemoglobinopathy groups RBC: red blood cell; hematocrit: packed cell volume; RDW: red cell distribution width; HbA: hemoglobin A; HbA₂: hemoglobin A₂; HbF: fetal hemoglobin; HbS: hemoglobin S HbS values represent pooled results from sickle hemoglobin disorders within the hemoglobinopathy group.

Parameter	Normal (Mean ± SD)	Hemoglobinopathy (Mean ± SD)	t-value	p-value
Hemoglobin	10.08 ± 0.52	9.32 ± 1.19	7.142	<0.001
RBC	3.73 ± 0.46	3.99 ± 0.62	-4.038	<0.001
Hematocrit	30.63 ± 1.96	29.04 ± 4.38	3.744	<0.001
RDW	15.13 ± 1.66	18.98 ± 4.17	-10.500	<0.001
HbA	95.02 ± 1.90	50.33 ± 27.31	20.325	<0.001
HbA₂	2.51 ± 0.44	3.21 ± 0.84	-8.961	<0.001
HbF	1.22 ± 0.49	7.90 ± 9.41	-8.826	<0.001
HbS	0.00	37.75 ± 20.86	-22.540	<0.001

Correlation analysis of hemoglobin fractions and hematological parameters

Correlation analysis demonstrated no significant association between HbA₂ and MCV or MCH. HbF showed a significant negative correlation with hemoglobin concentration (r = −0.558, p <0.001). HbS levels demonstrated a strong positive correlation with RDW (r = +0.735, p <0.001) and a strong negative correlation with hemoglobin concentration (r = −0.612, p <0.001) (Table 5).

**Table 5:** Correlation of hemoglobin fractions with hematological parameters MCV: mean corpuscular volume; MCH: mean corpuscular hemoglobin; RDW: red cell distribution width; HbA: hemoglobin A; HbA₂: hemoglobin A₂; HbF: fetal hemoglobin; HbS: hemoglobin S

Variables Compared	Pearson r	p-value	Interpretation
HbA₂ vs MCV	+0.020	0.745	Not significant
HbA₂ vs MCH	+0.006	0.926	Not significant
HbF vs Hemoglobin	–0.558	<0.001	Significant negative
HbS vs RDW	+0.735	<0.001	Strong positive
HbS vs Hemoglobin	–0.612	<0.001	Strong negative

ROC curve and AUC analysis of red cell parameters

**Figure 2:**
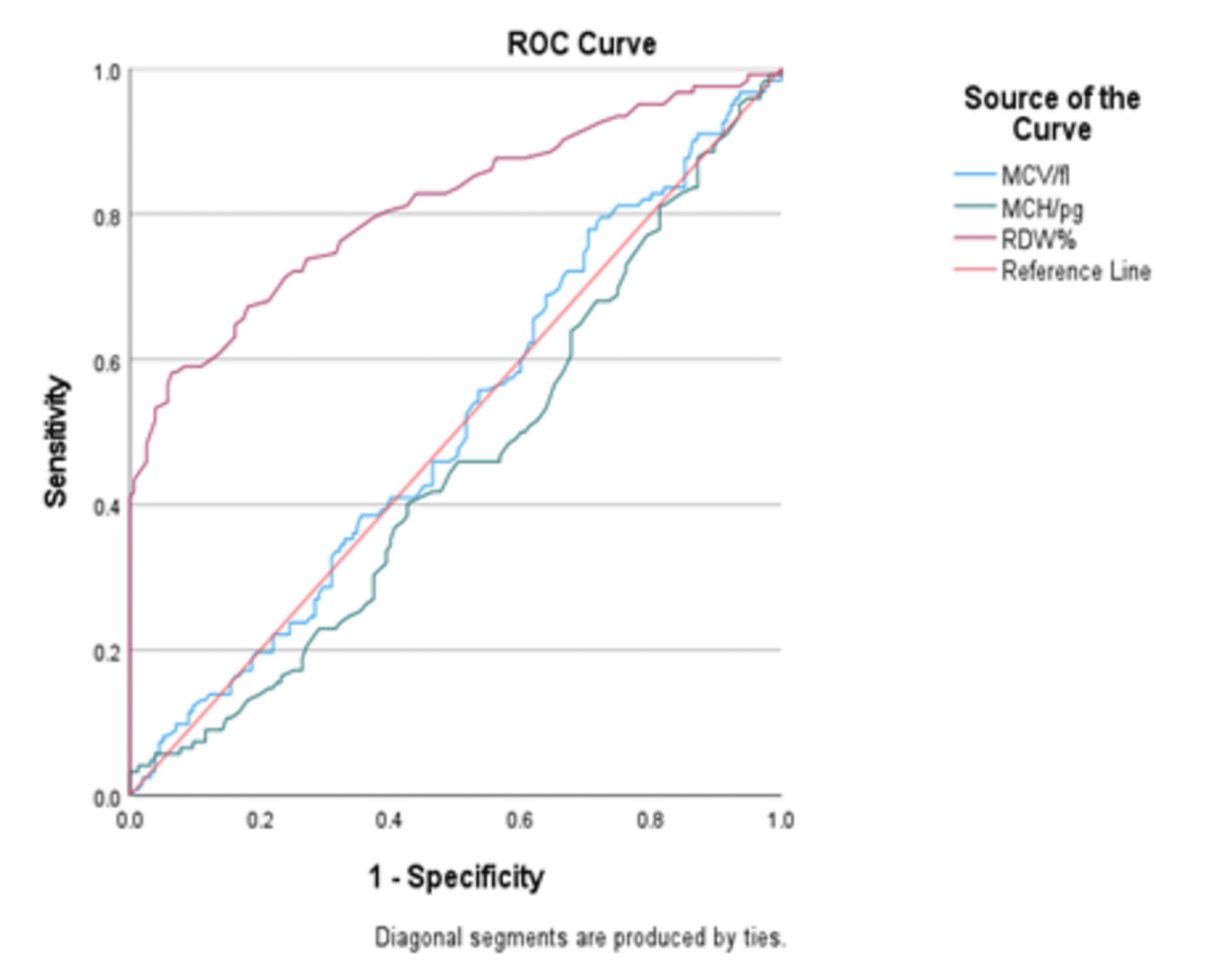
ROC curve of red cell parameters for predicting hemoglobinopathies MCV: mean corpuscular volume; MCH: mean corpuscular hemoglobin; RDW: red cell distribution width; ROC: receiver operating characteristic

ROC curve analysis demonstrated that RDW had significantly superior discriminative ability for predicting hemoglobinopathies compared to MCV and MCH. RDW (Figure 2). 

RDW showed the highest area under the curve (AUC of 0.811), indicating better discriminative ability (Table 6). At an optimal RDW cut-off of ≥16.75%, the sensitivity, specificity, positive and negative predictive values (PPV and NPV), and diagnostic accuracy for predicting hemoglobinopathies were 67.2%, 81.9%, 74.5%, 76.0%, and 75.5%, respectively.

**Table 6:** AUC analysis of red cell parameters MCV: mean corpuscular volume; MCH: mean corpuscular hemoglobin; RDW: red cell distribution width; AUC: area under the curve

Parameter	AUC	95% CI	p-value	Interpretation
MCV	0.510	0.441–0.578	0.779	Poor discrimination
MCH	0.454	0.386–0.522	0.190	No significant discrimination
RDW	0.811	0.758–0.864	<0.001	Good discrimination

## Discussion

Our study evaluated the frequency and hematological association of hemoglobinopathies among anemic females of reproductive age and found abnormal HPLC patterns in 122 (44%) participants. Although microcytic hypochromic anemia was the predominant morphological pattern observed, the study included all anemic women meeting the inclusion criteria and was not restricted to microcytic hypochromic anemia alone. Sickle cell trait was the most common abnormality, identified in 72 (26%) participants, followed by sickle cell disease in 30 (11%) and β-thalassemia trait in 19 (7%) participants. The findings demonstrate that hemoglobinopathies were frequently detected among anemic women referred for HPLC evaluation. Our study population comprised younger women, with 206 (74%) being less than 40 years old, similar to results by Agarwal et al. and Manger et al., who observed higher rates of hemoglobinopathies in women of reproductive age during antenatal screening [13-15]. In our study, the prevalence of hemoglobinopathies was higher compared to other studies done by Panchal (20.1%) [13] and Agarwal (17.8%) [14]. This difference could be a regional variation since the prevalence of hemoglobin variants in eastern India is comparatively high, particularly HbS, as reported by Ghosh et al. and Madan et al. [16,17]. The high frequency of abnormal HPLC patterns in our cohort also highlights the diagnostic overlap between IDA and inherited hemoglobin disorders in routine clinical practice.

As observed, the RBC count of participants with β-thalassemia trait was significantly higher than that of non-β-thalassemia trait individuals, while the MCV value was significantly lower than that of the non-β-thalassemia trait, which was consistent with the classical microcytic hypochromic picture reported by Adeyemo et al. and Schechter et al. [18,19]. However, the levels of HbA₂ showed overlap with non-β-thalassemia trait cases, which were not the usual diagnostic expectations. Coexistence with iron deficiency is a possible explanation that may down-regulate HbA2 levels, as observed by Mutua et al. [20]. As the iron analysis was not done in our study, the contribution of concurrent iron deficiency could not be fully evaluated. Hemoglobin levels were found to be significantly low in sickle cell disease cases and markedly high in RDW cases, with HbS and HbF fractions. The protective effect of high HbF on the severity of the disease was in concordance with the study done by Schechter et al. [19]. We also noted that HbS was inversely correlated with hemoglobin concentration and positively correlated with RDW, which lends further support to the relationship of hemolysis, anisocytosis, and the severity of the disease.

RDW had the greatest discriminatory power and was significantly elevated in both sickle cell trait and sickle cell disease groups among the routine hematological parameters. MCV and MCH were less helpful in the discrimination of hemoglobinopathy subgroups and had low diagnostic specificity as noted by Shah et al. [21]. Our study additionally showed that the predictive power of RDW over MCV and MCH was superior, although the use of RDW as a diagnostic marker was limited. HbA₂ correlated poorly with MCV and MCH, in contrast to the report of Ntaios et al. [22]. This discrepancy can be attributed to mixed anemia patterns, including the confounding effect of coexistent iron deficiency on standard hematological associations. Further, comparison of normal and abnormal HPLC groups showed significantly lower levels of hemoglobin and higher RDW in those with hemoglobinopathies, which is similar to reports by Shah et al. [21].

The strengths of our study include an adequate sample size (n = 277), focus on women of reproductive age, and the use of HPLC analysis for accurate identification of hemoglobinopathies. The inclusion of hematological parameters and their diagnostic associations, along with the regional focus, provides valuable epidemiological data from eastern India, which is endemic for hemoglobinopathies. However, the cross-sectional and single-center design of the study limits its generalizability. In addition, the lack of molecular evaluation and iron profile assessment limited the analysis of rare variants and the influence of coexisting conditions such as iron deficiency. The absence of serum ferritin, iron profile, vitamin B12, and folate assessment limits the evaluation of coexisting nutritional deficiencies and their contribution to hematological abnormalities. Since participants were recruited from women referred for HPLC testing, the findings reflect the frequency of hemoglobinopathies within a referral population and should not be interpreted as prevalence estimates for the general population. Further large-scale multicentric studies incorporating advanced molecular and genetic techniques are required for a more comprehensive evaluation of these findings.

## Conclusions

Hemoglobinopathies were frequently identified among women of reproductive age suffering from anemia, with sickle cell trait being the most common anomaly observed by HPLC. While hematological parameters were helpful in making an initial assessment, there was significant overlap among the various causes of anemia. RDW showed higher discriminative power compared to other red cell indices. However, red cell parameters alone did not provide sufficient data to be considered a standalone diagnostic investigation for hemoglobinopathies. HPLC provided better detection and characterization of hemoglobin variants and may serve as a useful tool for targeted assessment in high-risk populations. Although HPLC improved detection and characterization of hemoglobin variants, comprehensive evaluation, including iron profile and other nutritional parameters, remains essential for accurate assessment of anemia in women of reproductive age.
